# Antioxidant and DNA Damage Protecting Activity of Exopolysaccharides from the Endophytic Bacterium *Bacillus cereus* SZ1

**DOI:** 10.3390/molecules21020174

**Published:** 2016-02-04

**Authors:** Li Ping Zheng, Tin Zou, Yan Jun Ma, Jian Wen Wang, Yu Qing Zhang

**Affiliations:** 1 Department of Horticulture, School of Architecture, Soochow University, Suzhou 215123, China; lpzheng@suda.edu.cn; 2 College of Pharmaceutical Sciences, Soochow University, Suzhou 215123, China; zouting0723@163.com (T.Z.); mdsdyx@126.com (Y.J.M.); 3 School of Biology & Basic Medical Sciences, Soochow University, Suzhou 215123, China

**Keywords:** *Artemisia annua* L., *Bacillus cereus*, exopolysaccharide, radical scavenging activity, oxidative damage

## Abstract

An endophytic bacterium was isolated from the Chinese medicinal plant *Artemisia annua* L. The phylogenetic and physiological characterization indicated that the isolate, strain SZ-1, was *Bacillus cereus*. The endophyte could produce an exopolysaccharide (EPS) at 46 mg/L. The 1,1-diphenyl-2-picrylhydracyl (DPPH) radical scavenging activity of the EPS reached more than 50% at 3–5 mg/mL. The EPS was also effective in scavenging superoxide radical in a concentration dependent fashion with an EC_50_ value of 2.6 mg/mL. The corresponding EC_50_ for scavenging hydroxyl radical was 3.1 mg/mL. Moreover, phenanthroline-copper complex-mediated chemiluminescent emission of DNA damage was both inhibited and delayed by EPS. The EPS at 0.7–1.7 mg/mL also protected supercoiled DNA strands in plasmid pBR322 against scission induced by Fenton-mediated hydroxyl radical. The preincubation of PC12 cells with the EPS prior to H_2_O_2_ exposure increased the cell survival and glutathione (GSH) level and catalase (CAT) activities, and decreased the level of malondialdehyde (MDA) and lactate dehydrogenase (LDH) activity in a dose-dependent manner, suggesting a pronounced protective effect against H_2_O_2_-induced cytotoxicity. Our study indicated that the EPS could be useful for preventing oxidative DNA damage and cellular oxidation in pharmaceutical and food industries.

## 1. Introduction

Free radicals such as superoxide, hydroxyl radical and other reactive oxygen species (ROS) are associated with cellular necrosis and a variety of pathological conditions such as cancer, degenerative disease in neurons, hepatopathies, antherosclerosis, and even aging [1]. Antioxidants, scavenging free radicals, are known to play important roles in preventing the ROS-linked diseases [2]. As many of the synthetic antioxidants used nowadays such as butylated hydroxyanisole and butylated hydroxytoluene are suspected to have cytotoxicity [3], growing attention has been paid to the development of efficient and safe antioxidants from natural resources [4,5]. Recently some bacterial exopolysaccharides (EPS) have been demonstrated to possess potent antioxidant activities and applications as natural antioxidants [6,7]. As a less investigated microorganisms “hidden” within host plants, endophytes residing inside the healthy tissue of foliage, roots, stems and bark have been noted for their diversity of secondary metabolites including steroids, alkaloids, terpenoids and peptides [8]. It has been reported that endophyte metabolites including phenolic compounds and flavonoids can be a potential source of novel natural antioxidants [9,10]. Liu *et al.* reported for the first time, the capacity of the bacterium endophyte *Paenibacillus polymyxa* to produce EPS with strong scavenging activities on superoxide and hydroxyl radicals [11].

EPS are produced by various genera of bacteria and *Bacillus* spp. have been known to produce extra cellular polysaccharides [12]. *Bacillus licheniformis* can produce EPS with antiviral and immunoregulatory activities [13]. Although EPS from the probiotic bacteria *Bacillus* spp. displayed significant antioxidant activity [14], the antioxidant activities from endophytic *Bacillus* spp. have not been investigated yet. In continuation of our characterization of new bioactive metabolites from endophytic microbes [15,16] and antioxidant potential of fungal extracts [17], we have now investigated the antioxidant activities of EPS from *Bacillus cereus* SZ1 isolated from the stem of the anti-malarial medicinal plant *Artemisa annua* L. The EPS was evaluated for *in vitro* scavenging ability of 1,1-diphenyl-2-picrylhydracyl (DPPH) free radical, superoxide anion and hydroxyl radicals, and ability of reducing H_2_O_2_-induced oxidative damage in PC12 cells. The protective effects of the EPS on DNA damage induced by H_2_O_2_ and hydroxyl radical were also studied by chemiluminescence and DNA nicking assays, respectively.

## 2. Results

### 2.1. Strain Isolation, Biochemical and Molecular Characterization

**Figure 1:**
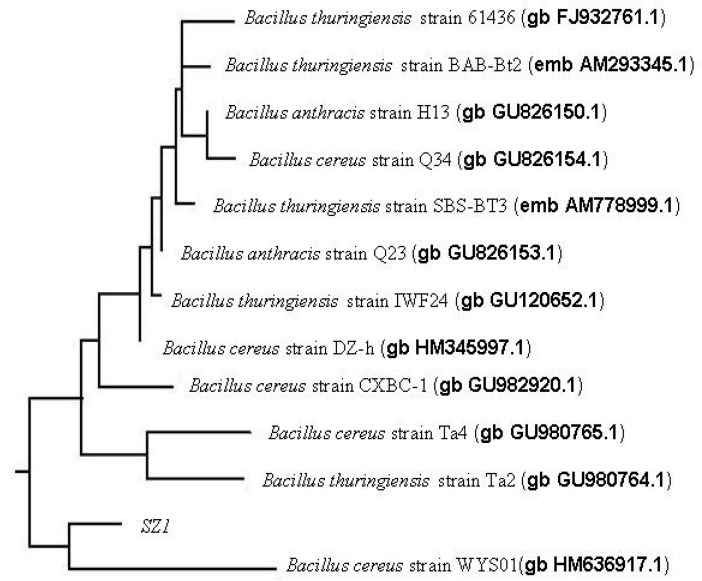
Phylogenetic tree based on the 16S rDNA sequences information of SZ1.

Isolate SZ1 was selected due to the higher 1,1-diphenyl-2-picrylhydracyl (DPPH) scavenging ability of its EPS in our preliminary experiments. The strain was Gram-positive, endospore-forming, motile, peritrichously flagellated with a straight rod-shape. The details of these and other morphological and physiological characteristics are summarized in Table 1. Furthermore, the 16S rRNA gene sequences of SZ1 (GenBank accession number HQ121399) were identified by polymerase chain reaction, sequencing and comparison to all sequences in GenBank. SZ1 was most closely aligned to *Bacillus* sp. with 99% similarity, mainly including *B. cereus*, *B.* *thuringiensis* and *B. anthracis*. Then, a phylogenetic tree constructed by the neighbor-joining method showed the closest match to that of *B. cereus* strain WYS01 (Figure 1). Therefore, based on the 16S rDNA sequence homology and the biochemical characterization we identified the isolate as a strain of *B. cereus* SZ1.

**Table 1:** Physiological and biochemical characteristics of strain SZ-1.

Characteristics	Result	Characteristics	Result
Gram staining	+ ^a^	d-glucose	+
Swelling spore	− ^b^	l-arabinose	−
Aerobic growth	+	d-xylose	+
Anaerobic growth	−	d-mannitol	+
Nitrate reduction	+	Lactose	+
Utilization of citrate	+	Raffinose	+
Voges-proskauer test	+	Cellobiose	+
Milk solidification	+	Indole production	−
Milk peptonization	+	Starch hydrolysis	+
Catalase	+	Indole production	−
Oxidase	+	Gas from glucose	+
urease	−	Growth temperature range	10–40 °C
Contactwise	+	Tolerance to NaCl	<10%

^a^: Positive; ^b^: Negative.

### 2.2. Growth Curve and EPS Production

**Figure 2:**
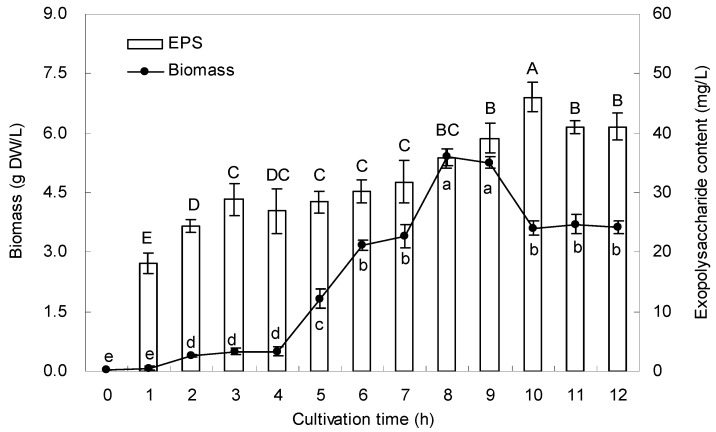
Time courses of bacteria biomass and EPS content in liquid cultures of *Bacillus cereus* SZ1. Data are mean ± S.D. of three replicates. The letters indicate the significant difference (*p* < 0.05) between the data of the different cultivation time.

The typical time course for the biomass and EPS production by SZ1 was examined in shake flask cultures (Figure 2). The biomass showed a lag-phase in the first 1–4 h and exponential growth in the next 4–8 h, then reached a maximum amount of 5.4 g dry weight (DW)/L after 8 h. The maximal production of EPS (46 mg/L) was obtained after 10 h. Then the level of EPS production became quite stable.

### 2.3. Chemical Analysis of EPS

**Figure 3:**
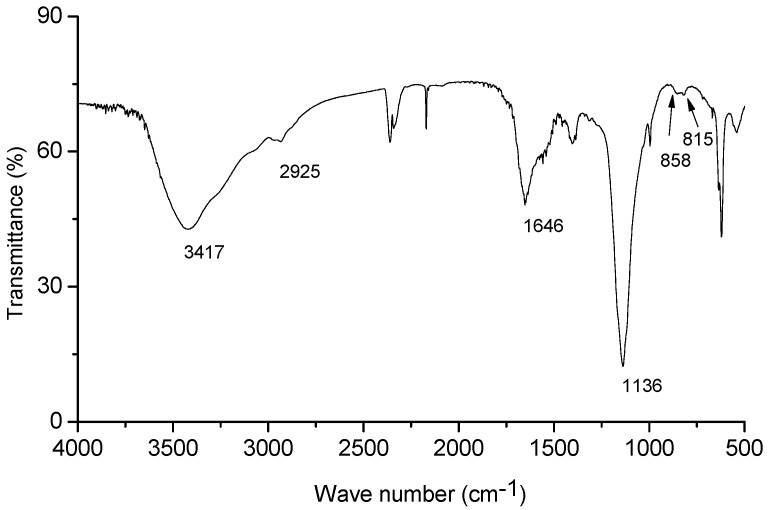
The infrared spectrum of the exopolysaccharide.

Figure 3 presents the IR spectrum of the crude EPS. The broad and intense stretching at 3417 cm^−1^ is characteristic of hydroxyl groups and the weak stretching at 2925 cm^−1^ is attributed to the C-H bonds [18]. The absorption bands at 1136 cm^−1^ confirmed that the EPS is an acidic polysaccharide [19]. The band at 1646 cm^−1^ can be attributed to water bound to the phospholipid molecule [20]. The quantitative estimation showed that the EPS contained 77.75% of total sugars and 15.56% of total protein. However, saponins, total phenols, tannins and steroids were not found in EPS.

### 2.4. Scavenging Effect on DPPH Radicals

**Figure 4:**
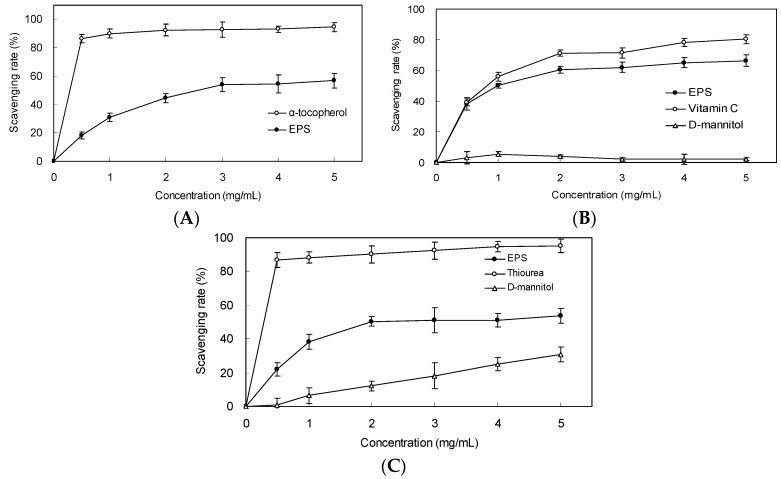
Scavenging effect on (**A**) DPPH radical, (**B**) superoxide anion and (**C**) hydroxyl radical by EPS from *Bacillus cereus* SZ1. α-tocopherol was used as positive control in DPPH radical scavenging test (**A**); vitamin C was used as positive control in the superoxide anion scavenging test (**B**); thiourea was used as positive control in hydroxyl radical scavenging test (**C**). Values represent the mean ± S.D. of triplicate samples.

To investigate the effects of EPS on the antioxidant activity *in vitro*, the DPPH scavenging rate of the EPS was examined. As shown in Figure 4A, the scavenging effect increased with the increasing EPS concentrations from 0.5 to 5.0 mg/mL. At 3.0 mg/mL, as much as 54.0% of DPPH radical was scavenged by the EPS.

### 2.5. Scavenging Effect on Superoxide Anion and Hydroxyl Radicals

The superoxide radical was generated in a phenazine methosulphate (PMS)/nicotinamide adenine dinucleotide-reduced (NADH) system and assayed by the reduction of nitroblue tetrazolium (NBT). As shown in Figure 4B, the EPS scavenged superoxide radical in a concentration-dependent manner. At 1.0 mg/mL, as much as 50.1% of superoxide radical was scavenged by the EPS. The EC_50_ value of the EPS for eliminating superoxide radical was 2.6 mg/mL. The EC_50_ of ascorbic acid as a positive control detected in the same experimental procedure was 2.3 mg/mL.

The hydroxyl radical scavenging activity of the EPS was assessed using deoxyribose assays. The EPS and mannitol exhibited scavenging activity on hydroxyl radical in a dose-dependent manner (0.5–5.0 mg/mL) (Figure 4C). Regarding the magnitude of the action, the suppressive effect of the EPS on deoxyribose damage was greater than that of mannitol. At 2.0 mg/mL, the scavenging activities of the EPS and mannitol against hydroxyl radical were 50.4% and 12.2%, respectively.

### 2.6. Reducing Power

**Figure 5:**
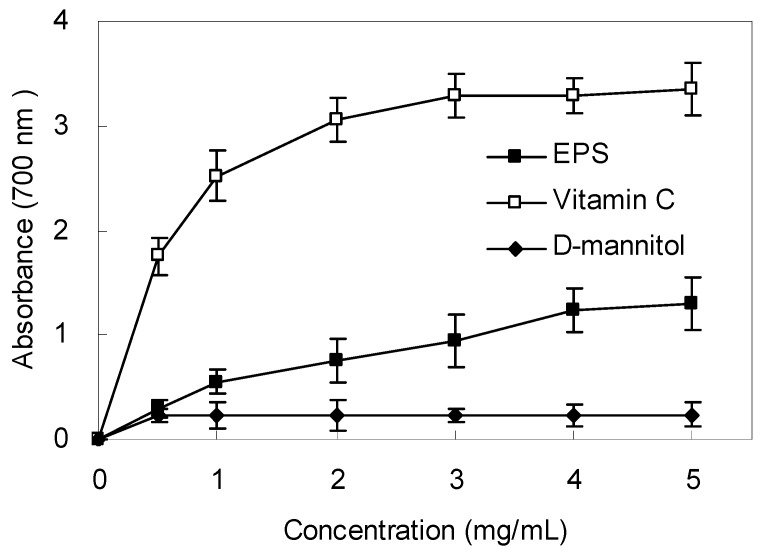
Reducing power of EPS from *Bacillus cereus* SZ1. Ascorbic acid was used as positive control. Values represent the mean ± S.D. of triplicate samples.

The reducing power of the EPS is shown in Figure 5. Like the other antioxidant activities, the reductive potential of EPS exhibited a dose-dependent activity within a concentration range of 0.5–5.0 mg/mL. However, the reducing power was lower than that of ascorbic acid, which indicated that the EPS had moderate reducing power. There was almost no significant reducing power of the mannitol solution at the same concentration as to the samples.

### 2.7. DNA Protective Effect

**Figure 6:**
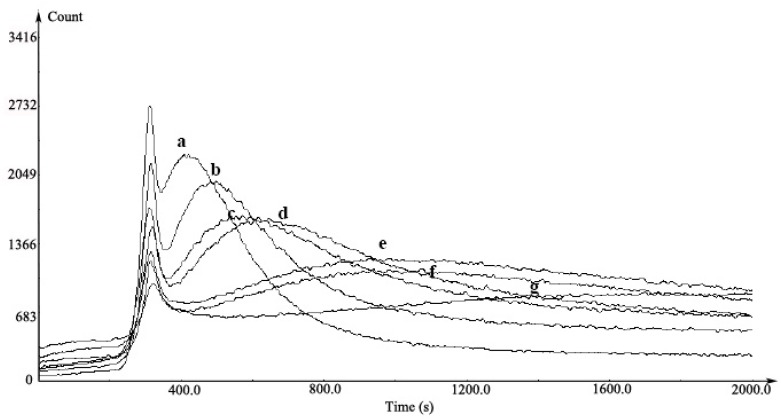
The inhibition of EPS from *Bacillus cereus* SZ1 on chemiluminescence induced by DNA damage. a, reaction mixture (copper sulfate, 0.05 mM; 1,10-phenanthroline, 0.35 mM; ascobate acid; 0.35 mM in pH 5.2 NaOAc/HOAc buffer (0.1 M) and 0.5% H_2_O_2_) with 1 μg/mL DNA; b–g, reaction mixture containing 1 μg/mL DNA with the EPS at 0.167, 0.333, 0.667, 1.000, 1.333 and 1.667 mg/mL, respectively.

The protective effect of different concentrations of EPS samples on damaged DNA in the chemiluminescence system is shown in Figure 6. In this system, an initial small peak was generated via phenanthroline attacked by •OH produced in the Fenton reaction. The second was a lag peak that had DNA damage by hydroxide radical. The light emission (curve a) showed the level of DNA damage by the phenanthroline-copper complex and H_2_O_2_ after the addition of DNA to the solution. An inhibited and delayed light emission (curves b–g) was observed after the treatment of EPS at 0.17–1.67 mg/mL. Our results suggested that the DNA damage induced by ROS could be reduced significantly in the presence of the EPS.

**Figure 7:**
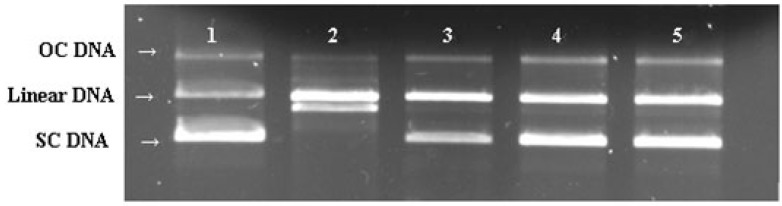
Protective effect of the exopolysaccharide on plasmide DNA breaks by •OH generated from a Fenton reaction: (lane 1) DNA incubated without Fenton reactant; (lane 2) DNA incubated with Fenton reactant; (lane 3–5) DNA incubated with Fenton reactant and the EPS at 0.667, 1.167 and1.667 mg/mL, respectively.

The DNA protective effect of the EPS was also evaluated using a broken DNA assay (Figure 7). DNA derived from plasmid showed three bands on agarose gel electrophoresis (line 1), in which the prominent faster moving band corresponded to the native supercoiled circular DNA (SC DNA), the second band referred to linear DNA and the slowest moving was the open circular form (OC DNA). Compared with plasmid DNA control (Line 1 of Figure 7), the SC DNA was completely converted to the linear form due to hydroxyl radical-induce DNA damage (Line 2 of Figure 7). This damage can be reduced in the presence of EPS and SC DNA was restored from other forms to its original supercoiled form.

### 2.8. Protection of PC12 Cells from H_2_O_2_-Induced Injury

**Figure 8:**
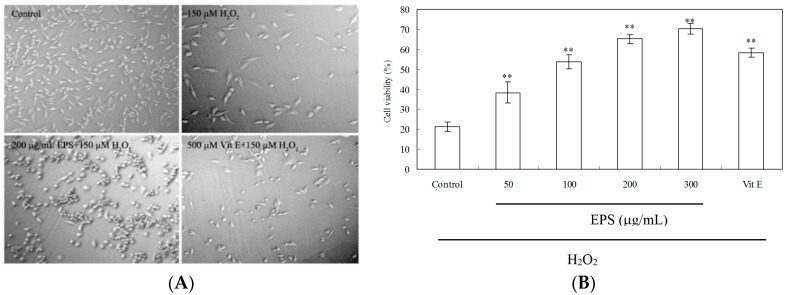
The protective effect of EPS on H**_2_**O**_2_**-induced damage in PC12 cells. (**A**) Cell morphology, Original magnification: ×150; Cells were pretreated with 50–300 μg/mL EPS or 500 μM vitamin E (Vit E) for 24 h. The control group was treated with fresh medium only under the same culture conditions. Cells were incubated in the presence of 150 μM H_2_O_2_ for 3 h; (**B**) Cell viability was determined by the MTT reduction assay. Data presented are the means ± SD of results from three independent experiments (** *p* < 0.01 *vs.* H_2_O_2_-treated control group).

As shown in Figure 8A, PC12 cells exhibited a significant decrease in cell number and cell damage after treatment with 150 μM H_2_O_2_ for 3 h. When cells were pretreated with EPS at 200 μg/mL or 500 μM vitamin E (Vit E) as the positive control for 24 h, H_2_O_2_-induced cytotoxicity was significantly mitigated. As estimated by 3-(4,5-dimethylthiazol-2-yl)-2,5-diphenyltetrazoliumbromide (MTT) assay (Figure 8B), H_2_O_2_ treatment caused a significant decrease of cell viability to 21.3%. EPS demonstrated the protective effect in a dose-dependent manner while incubation of PC12 cells with EPS (50–300 μg/mL) for 24 h significantly elevated the cell viability to a range of 38.4%–70.3%. The viability of H_2_O_2_-induced cells treated with Vit E (500 μM) was 58.3% only.

**Figure 9:**
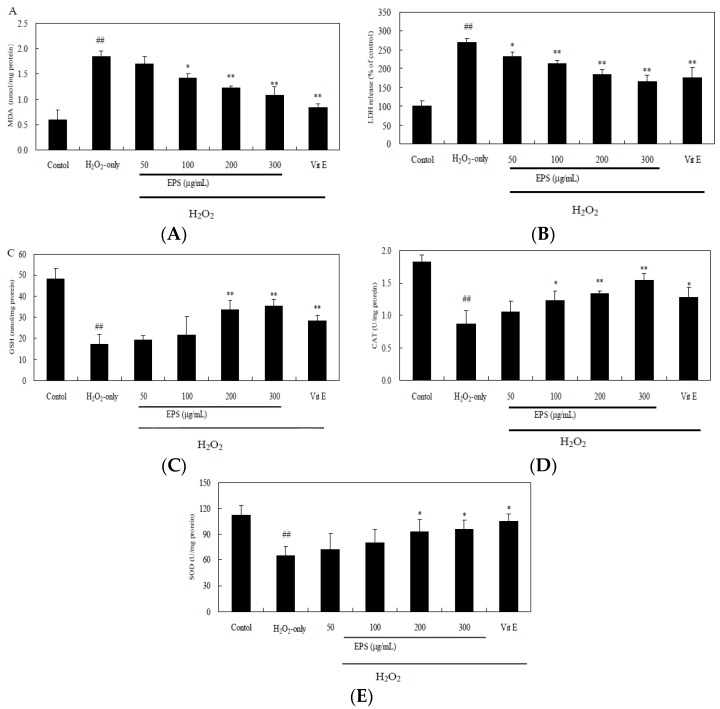
Effect of EPS on MDA (**A**); LDH (**B**); intracellular GSH level (**C**); CAT (**D**) and SOD (**E**) activity in PC12 cells. Cells were pretreated with 50–300 μg/mL EPS or 500 μM vitamin E (Vit E) for 24 h and then incubated in the presence of 150 μM H_2_O_2_ for 3 h. The control group was treated with fresh medium only under the same culture conditions. Data presented are the means ± SD of results from three independent experiments (## *p* < 0.01 *vs.* control; * *p* < 0.05 and ** *p* < 0.01 *vs.* H_2_O_2_-treated cells).

H_2_O_2_ also cause an increase of malondialdehyde (MDA) level and the release of lactate dehydrogenase (LDH), while pre-incubation of cells with EPS (100–300 μg/mL) attenuated markedly this increase (Figure 9A,B). Additionally, H_2_O_2_ exposure led to a decrease in glutathione (GSH) level, catalase (CAT) and superoxide dismutase (SOD) activity (Figure 9C–E). Such loss in H_2_O_2_-treated cells was rescued by EPS in a dose-dependent manner.

## 3. Discussion

Endophytic microbes, significant residents in healthy plants, have attracted more attention recently with their physiological role and bioactive metabolites [8]. The endophytic actinobacterial and fungi from the medicinal plant *A. annua* have been found to be capable of synthesizing a rich variety of metabolites with versatile bioactivities including herbicidal, antimicrobial activity and plant growth regulatory promoting activity [21,22]. Some endophytic *Bacillus* spp. have been found to be beneficial to their host plants, stimulating plant growth [23] and inducing resistance to plant pathogens [24]. Ryu *et al.* reported that two endophytic strains *B. subtilis* GB03 and *B. amyloliquefaciens* IN937a released the volatile components 2,3-butanediol and acetoin to trigger the greatest level of growth promotion of *Arabidopsis thaliana* [25]. To the best of our knowledge, this is the first report on endophytic *Bacillus* in *A. annua*. In our present research, crude EPS of endophytic *Bacillus* cereus SZ1 were found to possess the antioxidant activity. Pavlo *et al.* that plant colonization by endophytic bacteria could modulate the activity of plant antioxidant system as an early event in the development of the plant defense response [26]. Although the EPS from endophytic *Bacillus* possessed the antioxidant activity *in vitro*, our results suggested a possibility that the endophyte could change the cellular redox status by released EPS and is most probably associated with the strong adaptability and competitiveness of host *A. annua* in nature [27].

There are a very few reports on the synthesis of EPS and their antioxidant activities from *Bacillus* strains. A soil *B. subtilis* strain could synthesized the bioflocculant EPS [28]. The production of *Bacillus* EPS was found to be potentially of importance in sewage treatment processes for the removal of toxic heavy metal pollutants [29]. The EPS of four different monosaccharides (glucose, fucose, galactose and mannose) produced by *B. coagulans* had significant antioxidant and free radical scavenging activities [30]. In this study, it is the first time to report that EPS from endophytic *Bacillus* in scavenging superoxide and hydroxyl radicals in a concentration dependent fashion with EC_50_ values of 2.6 and 3.1 mg/mL, respectively. Although compared with the positive control (vitamin C or thiourea) the scavenging abilities of the EPS are moderate, EC_50_ values established for the EPS are lower than those from *B. licheniformis* KS-17 and *B. licheniformis* KS-20 [31]. It was been suggested that the antioxidant capacity of EPS may be ascribed to its hydroxyl group characterized at 3417 cm^−1^ IR spectrum (Figure 3), which donates electrons to scavenge the radicals. The protein residues (15.56%) in EPS also need to be further analyzed to account for its radical scavenging activities. Compositionally, EPS were found to consist of different monosaccharides including galactose, glucose, rhamnose, mannose and glucuronic acid. These monosaccharides except for glucuronic acid are effective reductive agents as they have a hidden aldehyde moiety [32]. The radical scavenging activity of EPS may be based on the reductive nature of these monosaccharides. A great number of studies showed that purified polysaccharides from crude polysaccharides were more effective on antioxidant ability *in vitro* than crude polysaccharides [6]. Hence, the purification of the crude EPS from endophytic *Bacillus* and the molecular composition, antioxidant activities of the purified fractions need further investigation.

There is a considerable amount of evidence revealing an association between DNA damage and some human health problem, such as cancer and aging [33]. Many microbial polysaccharides are known to exhibit an effective protection against the DNA damage [34]. Ma *et al.* demonstrated that oxidation of DNA by phenanthroline-copper/ascorbate/H_2_O_2_ emitted light in a concentration-dependent manner [35]. They grouped antioxidants as inhibiting type and delaying type according to their difference in effects on the luminescence. In their case of inhibiting type of antioxidants such as tanshinone II-A, chemiluminescent yield was decreased with increasing concentration of the antioxidant. The effect of trolox (delaying type) was demonstrated as the luminescence peak was delayed and the interval became longer and longer with increasing trolox concentration. In our results, the EPS presented both inhibiting and delaying activity (mixed type) (Figure 6), similar to the effects of green tea polyphenols [35]. Moreover, in our study, the protective effect against DNA strand scission induced by Fenton-mediated hydroxyl radical was also investigated (Figure 7). The hydroxyl radical generated by Fenton reactant could attack DNA and resulted in a dramatic scission of supercoiled (SC) DNA strand to open circular (OC) or linear DNA [36]. In our research, the result of the cleavage of SC DNA to give prominent linear DNA due to Fenton reactant was changed, confirming the scavenging activity of EPS against hydroxyl radical (Figure 4).

To explore further the potential mechanism of antioxidant activity of EPS, we examined the protective effect of EPS on H_2_O_2_-induced cell damage in PC12 cells. H_2_O_2_, generating detrimental hydroxyl radicals as the major component of ROS, has been extensively used as an inducer of oxidative stress in PC12 cell which is commonly used as a reliable model for testing the prevention of ROS-induced neuronal death [37]. It has been reported that bacterial EPS-3 isolated from the culture of *L. planterum* LP6 and fungal EPS-2 from marine *Keissleriella* sp. had a notable protective effect against oxidative damage on PC12 cells [38,39]. In this study, treatment of PC12 cells with 150 μM H_2_O_2_ for 3 h induced an elevation of oxidative stress characterized by MDA production and LDH release (Figure 9). However, when PC12 cells were preincubated with EPS, the H_2_O_2_-induced reduction in cell survival was remarkably attenuated (Figure 8), suggesting a protective effect of EPS2. In addition to possible direct free radical scavenging, EPS may exert indirect effects, such as the modulation of CAT activity to detoxify H_2_O_2_ to oxygen and water (Figure 9D). Our study also showed that H_2_O_2_ caused the decrease in GSH content by 64.1%, however, pretreatment of PC12 cells by EPS (200–300 μg/mL) resulted in about 2-fold increase of GSH compared to the H_2_O_2_-treated cells (Figure 9C). As GSH serves as an electron donor to unstable ROS and performs cell-protective antioxidant role [40], our result indicated that GSH metabolism could be regulated by EPS as another detoxifying system to prevent ROS-induced damages. Further studies will be needed to reveal the mechanism of EPS-induced cytoprotective effect in PC12 cells. This profound protective effect of the EPS against oxidative damage and moderate activity of free radical scavenging presented a clue that endophytic *Bacillus cereus* SZ1 may serve as an ideal candidate for readily exploitable natural antioxidants.

## 4. Experimental Section

### 4.1. Reagents and Materials

Taq DNA polymerase, 10 × reaction buffer, MgCl_2_ (25 mM) were purchased from Fermentas (Ontario, ON, Canada); dNTP (10 mM) from Roche (Basel, Switzerland); DPPH, NADH, 2-deoxy-d-ribose, calf thymus DNA, α-tocopherol, agarose and ethidium bromide (EtBr) from Sigma-Aldrich (St. Louis, MO, USA); NBT and PMS from Amresco (Solon, OH, USA); Plasmid pBR322 DNA, 2-thiobarbituric acid (TBA), trichloroacetic acid (TCA), H_2_O_2_, MTT, Vit E, ascorbic acid and mannitol were from Sinopharm Chemical Reagent Company (Shanghai, China). Other chemicals and reagents were of commercially available analytical grade.

### 4.2. Isolation and Identification of Selected Endophytic Bacteria

Fresh stems of *A. annua* were collected from apparently healthy plants growing in the suburbs of Nanjing, China. A total of ten plants were collected. Picked plants were put into plastic pot and processed the following day. The stems were cut into rods (about 1 cm in length) and rinsed in running water. Surface sterilization followed described procedures [41]. After successive sterilization in 75% ethanol (20 s) and 2.5% sodium hypochlorite (10 min), the stem rods were rinsed three times in sterile distilled water to remove the disinfectant; samples were then dried with sterile filter paper and subsequently ground with 1 mL 0.9% sodium chloride with a sterile mortar, with sterile quartz being added to improve the wall disruption. Then 100 µL of tissue extracts were streaked onto a nutrient agar plate in triplicate. Aliquots (100 µL) of the last washing water were planted as sterility controls, which never showed to be contaminated. Plates were incubated at 30 °C for 48 h to isolate bacteria.

The physiological characteristics of the isolate strain SZ1 were characterized according to the procedures outlined in Bergey’s Manual of Systematic Bacteriology [42]. In addition, SZ1 strain was identified by determination of 16S rDNA gene sequences. Bacterial lysates were prepared by dissolving a bacterial colony in 20 µL sterile distilled water and incubation at 100 °C for 15 min; 2µL lysate was used for amplification reaction by polymerase chain reaction (PCR). Amplification of 16S rDNA was performed in a total volume of 50 µL containing 5 µL of 10 × reaction buffer, 3 µL MgCl_2_ (25 mM), 1 µL dNTP (10 mM), 0.25 µL Taq DNA polymerase (5 U/µL), 1 µL of each primer [27f, 5′-GAGAGTTTGATCCTGGCTCAG-3′, and 1525r, 5′-AAAGGAGGTGATCCAGCC-3′], corresponding to the *Escherichia coli* rDNA nucleotide positions where the 3′ end of the primer anneals in forward (f) or reverse (r) orientation. Thirty seven rounds of amplification were performed in a thermocycler (Bio-Rad, Hercules, CA, USA) at 95 °C (30 s) and 58 °C annealing (30 s) and 72 °C extension (40 s), with a final extension for 7 min. The 16S rDNA sequences obtained were matched against nucleotide sequences present in GenBank using the BLASTn program. For further characterization of SZ1, a neighbor-joining phylogenetic tree was constructed with the CLUSTAL W program [43].

### 4.3. Cultivation and Extraction of EPS

The strain was maintained on a solid Luria-Bertani (LB) medium with 15 g/L agar at 4 °C. Liquid fermentation was performed in a liquid medium containing (per liter) 10 g peptone , 5 g yeast extract, 10 g NaCl and initial pH at 7.0. Inoculum was prepared by transferring one loop full of culture from solid LB medium to an Erlenmyer flask (250 mL) containing 50 mL medium. The seed cultures were grown at 30 °C in shake flasks at 180 rpm for 7 h, then 10 mL of the seed culture was transferred into a 250 mL Erlenmyer flask containing 100 mL medium under shaking (180 rpm) for 10 h.

The fermentation broth was centrifuged at 6000 rpm for 10 min. The liquid phase was condensed to 1/3 volume, mixed with three volumes of 95% ethanol (*v*/*v*), and maintained at 4 °C overnight for precipitation. The precipitate was collected (after centrifugation at 7000 rpm for 10 min) and washed with absolute ethanol, and then redissolved in distilled water and followed by deproteinization with 1/5 volume of Sevag reagent (CHCl_3_-BuOH, *v*/*v* = 5/1) for seven times [44]. The deproteinized solution was then dialyzed against distilled water, concentrated, lyophilized to obtain the crude EPS.

### 4.4. Chemical Analysis of EPS

Spectra of EPS were determined using an IR spectrophotometer (ProStar LC240, Varian, Palo Alto, CA, USA) with KBr pellets in the range 4000–400 cm^−1^ [20]. The presence of various phytoconstituents such as sulfate, saponins, steroids, total phenols and tannins were analyzed qualitatively using standard protocols [45]. Further total sugars and proteins in EPS were quantified with methods by Hedge and Hofrreiter [46], and the Bradford method [47], respectively.

### 4.5. Radical Scavenging Property and Antioxidant Activity in Vitro

#### 4.5.1. DPPH Radical Scavenging Assay

DPPH radical scavenging activity was done according to the method of Hatano *et al.* [48] with some modifications. In brief, 1 mL of DPPH (Sigma) solution (0.2 mM in 95% ethanol (*v*/*v*)) was incubated with different concentrations of the EPS. The reaction mixture was shaken and incubated at room temperature in dark for 30 min and then the absorbance at 517 nm was followed spectrophotometrically. α-Tocopherol was used as a positive control and the sample solution without DPPH as a sample blank. The radical scavenging activity was determined as a decrease in the absorbance of DPPH and calculated according to the following equation:

Scavenging effect (%) = [A*_b_* − (A*_s_* − A*_sb_*)]/A*b* × 100

where A*_b_,* A*s* and A*_sb_* are the absorbances at 517 nm of the blank, sample or positive control, and sample blank respectively.

#### 4.5.2. Superoxide Radical Scavenging Assay

The superoxide radical scavenging ability of the extract was measured as described in our previous work [49]. The reaction mixture, contained different concentrations of the EPS, 10 μM PMS, 78 mM NADH and 50 μM NBT in Tris-HCl buffer (16 mM, pH 8.0), was incubated at room temperature for 5 min and the color reaction of the rest superoxide radical with NBT was detected at 560 nm. Ascorbic acid was used as a positive control and distilled water as a blank. The scavenging activity of superoxide radical (%) was thus calculated with the equation described as in the case of DPPH.

#### 4.5.3. Hydroxyl Radical Scavenging Assay

The deoxyribose method was used for determining the scavenging effect on hydroxyl racicals as described by Halliwell *et al.* [50]. EPS solution on varying concentration (0.5 mL) was added to 1.0 mL solution of 20 mM potassium phosphate buffer (pH 7.4) containing 2.8 mM 2-deoxy-d-ribose, 104 μM EDTA, 100 μM FeCl_3_, 100 μM ascorbic acid and 1 mM hydrogen peroxide. The obtained mixture was incubated at 37 °C for 1 h followed by addition of 1 mL of 10% TCA containing 0.5% TBA and then boiled at 100 °C for 15 min. It was cooled in ice and absorbance was taken at 532 nm. Thiourea was used as a positive control. The scavenging activity of hydroxyl radical (%) was thus calculated with the equation described as in the case of DPPH.

#### 4.5.4. Measurement of Reducing Power

The reducing power of the extract was determined by the method of Ahmadi *et al.* [51] with some modification. EPS (0–10 mg/mL) in phosphate buffer (2.5 mL, 0.2 mol/L, pH 6.6) were added to potassium ferricyanide (2.5 mL, 10 mg/mL), and the mixture was incubated at 50 °C for 20 min. Then trichloroacetic acid (2.5 mL, 10 mg/mL) was added to the mixture and then the mixture was centrifuged at 1160 *g* for 10 min. The supernatant (2.5 mL) was mixed with 2.5 mL of deionized water and ferric chloride (0.5 mL, 1.0 mg/mL).Then the absorbance was measured at 700 nm. A higher absorbance of the reaction mixture indicated a greater reducing power. Ascorbic acid and mannitol were used as controls.

#### 4.5.5. Chemiluminescence Assay for H_2_O_2_-Induced DNA Damage

DNA chemiluminescence was measured in phenanthroline-copper system by the method of Ma *et al.* [35]. 0.5 mM copper sulphate (0.1 mL), 3.5 mM 1,10-phenanthroline (0.1 mL), 10 μg/mL calf thymus DNA (0.1 mL), 3.5 mM ascobate (0.1 mL) and 0.1 mL EPS solution were premixed in 0.5 mL NaOAc/HOAc buffer (0.1 M, pH 5.2) and incubated at 18 °C for 5 min. Following this, 3% H_2_O_2_ was added to the solution to give a final volume of 1.2 mL. The kinetic curve of chemiluminescence produced in the phenanthroline-copper/H_2_O_2_/ascorbate system was immediately recorded with a computerized high-sensitivity single-photon counter (BPCL-2-KGC, the Institute of Biophysics, Beijing, China). The voltage in the photomultiplier was kept at 875 V.

#### 4.5.6. DNA Nicking Assay for Hydroxyl Radical Scavenging Activity

The method of hydroxyl radical-induced DNA breakage in plasmid pBR322 was modified from Kitts *et al.* [51]. Briefly, the experiment was performed in a microfuge tube containing 0.5 μL of pBR 322 plasmid DNA (200 μg/mL) in 3 μL of 50 mM potassium phosphate buffer (pH 7.4), 3 μL of 1 mM FeSO_4_, 2 μL of different concentrations of EPS and 4 μL of 8.8 M H_2_O_2_. The mixture was incubated at 37 °C for 30 min and then subjected to 0.8% (*w*/*v*) agarose gel electrophoresis. The gel was stained with 0.5 μg/mL ethidium bromide and then photographed under UV light.

### 4.6. Culture of PC12 Cells and Viability Assay

The rat pheochromocytoma PC12 cell lines were purchased from the Chinese Type Culture Collection (Shanghai Institute of Cell Biology, Chinese Academy of Science, Shanghai, China). PC12 cells were cultured in RPMI 1640 medium (pH 7.4) with 10% calf serum at 37 °C under 5% CO_2_. All experiments were carried out 24–48 h after cells were seeded in 96-well microplate. In all experiments, cells were pretreated for 24 h with indicated concentrations of EPS or 500 μM vitamin (Vit) E as a positive control. 150 µM H_2_O_2_ was added to the medium for 3 h. The induced damage by H_2_O_2_ was halted by replace of the H_2_O_2_-treated medium with the fresh medium. Assays for cell viability, lipid peroxidation and antioxidant enzyme activities were performed 24 h after cultured in fresh medium. The cell viability was measured in 96-well plates by MTT method as described by Li *et al.* [39]. The absorbance was detected at 570 nm with a microplate reader (KLx808, Bio-Tek, Winooski, VT, USA). Cell viability is expressed as a percentage of that of the control culture. The control group cells were only treated with fresh medium containing no H_2_O_2_ and EPS.

### 4.7. MDA, LDH, GSH, CAT and SOD Assays

PC12 cells in 96-well plates were cultured and treated according to the procedures described above. The harvested cells were washed two times with phosphate buffered saline (PBS) (0.1 M, containing 0.05 mM EDTA), then scraped into ice-cold PBS and homogenized. The homogenate was centrifuged at 4 °C at 10,000× *g* for 30 min. The resulting supernatant was stored at −20 °C until MDA and enzyme assays. The content of MDA was determined by using TBA method [38]. The CAT, SOD activity, the content of GSH and the leakage of LDH were determined using Assay Kits (Institute of Biological Engineering of Nanjing Jiancheng, Nanjing, China) according to the protocols.

### 4.8. Statistical Analysis

Experimental values were expressed as means ± standard deviation (SD) of triple separate experiments and subjected to the one-way ANOVA followed by Duncan’s multiple range test with the SPSS 11.0 (SPSS Inc., Chicago, IL, USA). Differences in means were considered to be significant for *p*-values < 0.05. A Student’s *t-*test was used for statistical comparisons of two means.

## 5. Conclusions

In this study, an endophytic *Bacillus* strain (SZ1), isolated from the medicinal plant *A. annua*, produced crude EPS at 46 mg/L. From 16S rDNA analysis, strain SZ1 was related to *B. cereus*. The EPS exhibited moderate scavenging activity on superoxide, hydroxyl radical and DPPH radicals. Furthermore, the EPS exerted a significant protective effect against oxidative DNA damage *in vitro* and protected PC12 cells against oxidative damages induced by H_2_O_2_ through reduction of lipid peroxidation, improvement of CAT activity and GSH level in cells. This study suggests that the EPS from endophytic *B. cereus* SZ1 might contribute for potential application in functional foods as a natural antioxidant agent or a new therapeutic agent aiming at the treatment of oxidative damage-derived neurodegenerative disorders and cancers.

## References

[1] Pryor W.A. (1986). Oxy-radicals and related species: Their formation, lifetimes, and reactions. Annu. Rev. Physiol..

[2] Aruoma O.I. (1998). Free radicals, oxidative stress, and antioxidants in human health and disease. J. Am. Oil Chem. Soc..

[3] Velioglu Y.S., Mazza G., Gao L., Oomah B.D. (1998). Antioxidant activity and total phenolics in selected fruits, vegetables, and grain products. J. Agric. Food Chem..

[4] Denis M.C., Furtos A., Dudonné S., Montoudis A., Garofalo C., Desjardins Y., Delvin E., Levy E. (2013). Apple peel polyphenols and their beneficial actions on oxidative stress and inflammation. PLoS ONE.

[5] Giampieri F., Alvarez-Suarez J.M., Battino M. (2014). Strawberry and human health: Effects beyond antioxidant activity. J. Agric. Food Chem..

[6] Asker M.M.S., Ahmed Y.M., Ramadan M.F. (2009). Chemical characteristics and antioxidant activity of exopolysaccharide fractions from *Microbacterium terregens*. Carbohydr. Polym..

[7] Liu J., Luo J.G., Ye H., Sun Y., Lu Z.X., Zeng X.X. (2010). *In vitro* and *in vivo* antioxidant activity of exopolysaccharides from endophytic bacterium *Paenibacillus polymyxa* EJS-3. Carbohydr. Polym..

[8] Zhang H.W., Song Y.C., Tan R.X. (2006). Biology and chemistry of endophytes. Nat. Prod. Rep..

[9] Huang W.Y., Cai Y.Z., Xing J., Corke H., Sun M. (2007). A potential antioxidant resource: Endophytic fungi from medicinal plants. Econ. Bot..

[10] Liu X., Dong M., Chen X., Jiang M., Lv X., Yan G. (2007). Antioxidant activity and phenolics of an endophytic *Xylaria* sp. from *Ginkgo biloba*. Food Chem..

[11] Liu J., Luo J.G., Ye H., Sun Y., Lu Z.X., Zeng X.X. (2009). Production, characterization and antioxidant activities *in vitro* of exopolysaccharides from endophytic bacterium *Paenibacilluspolymyxa* EJS-3. Carbohydr. Polym..

[12] Vijayabaskar P., Babinastarlin S., Shankar T., Sivakumar T., Anandapandian K.T.K. (2011). Quantification and characterization of exopolysaccharides from *Bacillus subtilis* (MTCC 121). Adv. Biol. Res..

[13] Arena A., Maugeri T.L., Pavone B., Iannello D., Gugliandolo C., Bisignano G. (2006). Antiviral and immunoregulatory effect of a novel exopolysaccharide from a marine thermotolerant *Bacillus licheniformis*. Int. Immunopharm..

[14] Kodali V.P., Perali R.S., Sen R. (2011). Purification and partial elucidation of the structure of an antioxidant carbohydrate biopolymer from the probiotic bacterium *Bacillus coagulans* RK-02. J. Nat. Prod..

[15] Wang J.W., Wu J.H., Huang W.Y., Tan R.X. (2006). Laccase production by *Monotospora* sp., an endophytic fungus in *Cynodon dactylon*. Bioresour. Technol..

[16] Wang J.W., Zheng L.P., Zhang B., Zou T. (2009). Stimulation of artemisinin synthesis by combined cerebroside and nitric oxide elicitation in *Artemisia annua* hairy roots. Appl. Microbiol. Biotechnol..

[17] Gao L.W., Wang J.W. (2012). Antioxiant potential and DNA damage protecting activity of aqueous extract from *Armillaria mellea*. J. Food Biochem..

[18] Santhiya D., Subramanian S., Natarajan K.A. (2002). Surface chemical studies on sphalerite and galena using extracellular polysaccharides isolated from *Bacillus polymyxa*. J. Colloid. Interface. Sci..

[19] Ananthi S., Raghavendran H.R.B., Sunil A.G., Gayathri V., Ramakrishnan G., Vasanthi H.R. (2010). *In vitro* antioxidant and in vivo anti-inflammatory potential of crude polysaccharide from Turbinaria ornate (Marine brown alga). Food Chem. Toxicol..

[20] Yan J.K., Li L., Wang Z.M., Wu J.W. (2010). Structural elucidation of an exopolysaccharide from mycelial fermentation of a *Tolypocladium* sp. fungus isolated from wild *Cordyceps*
*sinensis*. Carbohydr. Polym..

[21] Lu H., Zou W.X., Meng J.C., Hu J., Tan R.X. (2000). New bioactive metabolites produced by *Colletotrichum* sp., an endophytic fungus in *Artemisia annua*. Plant Sci..

[22] Li J., Zhao G.-Z., Huang H.-Y., Qin S., Zhu W.-Y., Zhao L.-X., Xu L.-H., Zhang S., Li W.-J., Strobel G. (2012). Isolation and characterization of culturable endophytic actinobacteria associated with *Artemisia annua* L.. Anton. Leeuwenhoek.

[23] Palaniappan P., Chauhan P.S., Saravanan V.S., Anandham R., Sa T. (2010). Isolation and characterization of plant growth promoting endophytic bacterial isolates from root nodule of *Lespedeza* sp.. Biol. Fertil. Soils.

[24] Islam S.M.A., Math R.K., Kim J.M., Yun M.G., Cho J.J., Kim E.J., Lee Y.H., Yun H.D. (2010). Effect of plant age on endophytic bacterial diversity of balloon flower (*Platycodon grandiflorum*) root and their antimicrobial activities. Curr. Microbiol..

[25] Ryu C.-M., Farag M.A., Hu C.-H., Reddy M.S., Wei H.-X., Paré P.W., Kloepper J.W. (2003). Bacterial volatiles promote growth in *Arabidopsis*. Proc. Natl. Acad. Sci. USA.

[26] Pavlo A., Leonid O., Iryna Z., Natalia K., Maria P.A. (2011). Endophytic bacteria enhancing growth and disease resistance of potato (*Solanum tuberosum* L.). Biol. Control..

[27] Duke S.O. (1987). Artemisinin, constituent of annual wormwood (*Artemisia annua*), is a selective phytotoxin. Weed Sci..

[28] Patil S.V., Bathe G.A., Patil A.V., Patil R.H., Salunkea B.K. (2009). Production of bioflocculant exopolysaccharide by *Bacillus subtilis*. Adv. Biotech..

[29] Fusconi R., Godinho M.J.L. (2002). Screening for exopolysaccharide-producing bacteria from sub-tropical polluted groundwater. Braz. J. Biol..

[30] Kodali V.P., Sen R. (2008). Antioxidant and free radical scavenging activities of an exopolysaccharide from a probiotic bacterium. Biotechnol. J..

[31] Song Y.R., Song N.-E., Kim J.-H., Nho Y.-C., Baik S.-H. (2011). Exopolysaccharide produced by *Bacillus licheniformis* strains isolated from Kimchi. J. Gen. Appl. Microbiol..

[32] Sun C., Wang J.W., Fang L., Gao X.D., Tan R.X. (2004). Free radical scavenging and antioxidant activities of EPS2, an exopolysaccharide produced by a marine filamentous fungus *Keissleriella* sp. YS 4108. Life Sci..

[33] Wiseman H., Kaur H., Halliwell B. (1995). DNA damage and cancer: measurement and mechanism. Cancer Lett..

[34] Yen G.C., Shih P.H., Song T.Y., Tsai M.C. (2007). Antioxidant properties of water-soluble polysaccharides from *Antrodia cinnamomea* in submerged culture. Food Chem..

[35] Ma W.J., Cao E.H., Zhang J., Qin J.F. (1998). Phenanthroline-Cu complex-mediated chemiluminescence of DNA and its potential use in antioxidation evaluation. J. Photochem. Photobiol. B Biol..

[36] Qian Z.J., Jung W.K., Byun H.G., Kim S.K. (2008). Protective effect of an antioxidative peptide purified from gastrointestinal digests of oyster, *Crassostrea gigas* against free radical induced DNA damage. Bioresour. Technol..

[37] Kim D.S., Park S.Y., Kim J.K. (2001). Curcuminoids from *Curcuma longa* L. (Zingiberaceae) that protect PC12 rat pheochromocytoma and normal human umbilical vein endothelial cells from βA(1–42) insult. Neurosci. Lett..

[38] Sun C., Shan C.Y., Gao X.D., Tan R.X. (2005). Protection of PC12 cells from hydrogen peroxide-induced injury by EPS2, an exopolysaccharide from a marine filamentous fungus *Keissleriella* sp. YS4108. J. Biotechnol..

[39] Li J.-Y., Jin M.-M., Meng J., Gao S.-M., Lu R.-R. (2013). Exopolysaccharide from *Lactobacillus planterum* LP6, Antioxidation and the effect on oxidative stress. Carbohydr. Polym..

[40] Armstrong J.S., Steinauer K.K., Hornung B., Irish J.M., Lecane P., Birrell G.W., Peehl D.M., Knox S.J. (2002). Role of glutathione depletion and reactive oxygen species generation in apoptotic signaling in a human B lymphoma cell line. Cell Death Differ..

[41] Barzanti R., Ozino F., Bazzicalupo M., Gabbrielli R., Galardi F., Gonnelli C., Mengoni A. (2007). Isolation and characterization of endophytic bacteria from the nickel hyperaccumulator plant *Alyssum bertolonii*. Microb. Ecol..

[42] Sneath P.H.A. (1986). Endospore-forming gram-positive rods and cocci. Bergey’s Manual of Systematic Bacteriology.

[43] Thompson J.D., Higgins D.G., Gibson T.J. (1994). CLUSTAL W: Improving the sensitivity of progressive multiple sequence alignment through sequence weighting, position-specific gap penalties and weight matrix choice. Nucleic. Acids Res..

[44] Staub A.M. (1965). Removal of protein-Sevag method. Method Carbohyd. Chem..

[45] Harbone J.B. (1973). Phytochemical Methods.

[46] Hedge J.E., Hofreiter B.T., Whistler R.L., Be Miller J.N. (1962). Determination of total carbohydrate by anthrone reagent. Carbohydrate Chemistry.

[47] Bradford M.M. (1976). Rapid and sensitive method for the quantitation of microgram quantities of protein utilizing the principle of protein-dye binding. Anal. Chem..

[48] Hatano T., Edamatsu R., Mori A., Fujita Y., Yasuhara T., Yoshida T., Okuda T. (1989). Effects of the interaction of tannins with co-existing substances. VI. Effects of tannins and related polyphenols on superoxide anion radical, and on 1,1-diphenylperylhydrazyl radical. Chem. Pharm. Bull..

[49] Halliwell B., Gutteridge J.M.C., Aruoma O.L. (1987). The deoxyribose method: A simple test-tube assay for determination of rate constants for reactions of hydroxyl radicals. Anal. Biochem..

[50] Ahmadi F., Kadivar M., Shahedi M. (2007). Antioxidant activity of *Kelussia odoratissima* Mozaff in model and food systems. Food Chem..

[51] Kitts D.D., Wijewickreme A.N., Hu C. (2000). Antioxidant properties of a north American ginseng extract. Mol. Cell Biochem..

